# Vandetanib (Zactima, ZD6474) Antagonizes ABCC1- and ABCG2-Mediated Multidrug Resistance by Inhibition of Their Transport Function

**DOI:** 10.1371/journal.pone.0005172

**Published:** 2009-04-23

**Authors:** Li-sheng Zheng, Fang Wang, Yu-hong Li, Xu Zhang, Li-ming Chen, Yong-ju Liang, Chun-ling Dai, Yan-yan Yan, Li-yang Tao, Yan-jun Mi, An-kui Yang, Kenneth Kin Wah To, Li-wu Fu

**Affiliations:** 1 Research Department, State Key Laboratory of Oncology in South China, Cancer Center, SunYat-Sen University, Guangzhou, People's Republic of China; 2 Department of Medical Oncology, State Key Laboratory of Oncology in South China, Cancer Center, SunYat-Sen University, Guangzhou, People's Republic of China; 3 Department of Thoracic Surgery, State Key Laboratory of Oncology in South China, Cancer Center, SunYat-Sen University, Guangzhou, People's Republic of China; 4 Department of Head and Neck, State Key Laboratory of Oncology in South China, Cancer Center, SunYat-Sen University, Guangzhou, People's Republic of China; 5 School of Pharmacy, the Chinese University of Hong Kong, Hong Kong, People's Republic of China; Cairo University, Egypt

## Abstract

**Background:**

ABCC1 and ABCG2 are ubiquitous ATP-binding cassette transmembrane proteins that play an important role in multidrug resistance (MDR). In this study, we evaluated the possible interaction of vandetanib, an orally administered drug inhibiting multiple receptor tyrosine kinases, with ABCC1 and ABCG2 *in vitro*.

**Methodology and Principal Findings:**

MDR cancer cells overexpressing ABCC1 or ABCG2 and their sensitive parental cell lines were used. MTT assay showed that vandetanib had moderate and almost equal-potent anti-proliferative activity in both sensitive parental and MDR cancer cells. Concomitant treatment of MDR cells with vandetanib and specific inhibitors of ABCC1 or ABCG2 did not alter their sensitivity to the former drug. On the other hand, clinically attainable but non-toxic doses of vandetanib were found to significantly enhance the sensitivity of MDR cancer cells to ABCC1 or ABCG2 substrate antitumor drugs. Flow cytometric analysis showed that vandetanib treatment significantly increase the intracellular accumulation of doxorubicin and rhodamine 123, substrates of ABCC1 and ABCG2 respectively, in a dose-dependent manner (*P*<0.05). However, no significant effect was shown in sensitive parental cell lines. Reverse transcription-PCR and Western blot analysis showed that vandetanib did not change the expression of ABCC1 and ABCG2 at both mRNA and protein levels. Furthermore, total and phosphorylated forms of AKT and ERK1/2 remained unchanged after vandetanib treatment in both sensitive and MDR cancer cells.

**Conclusions:**

Vandetanib is unlikely to be a substrate of ABCC1 or ABCG2. It overcomes ABCC1- and ABCG2-mediated drug resistance by inhibiting the transporter activity, independent of the blockade of AKT and ERK1/2 signal transduction pathways.

## Introduction

Multidrug resistance (MDR), the ability of cancer cells to acquire resistance to a broad spectrum of structurally and functionally different anticancer drugs, is thought to be a major obstacle to the success of cancer chemotherapy. It is often mediated by overexpression of ATP-binding cassette (ABC) transporters that remove substrate drugs out of the cells against a concentration gradient with the use of energy from ATP hydrolysis [1], [2]. 48 different ABC transporters have been identified in the human genome and are divided into seven subfamilies (A–G) based on sequence similarities, among which, ABCB1/P-glycoprotein, ABCC1/MRP1, and ABCG2/BCRP are the most important members [3].

ABCC1, encoded by the ABCC1 gene on chromosome 16p13.1, was first identified from MDR human NSCLC cell line H69/ADR. It can transport a broad-spectrum of substrate anticancer drugs mainly conjugated to glutathione, glucuronate and sulfate, including vincristine and doxorubicin [4]–[6]. ABCG2, also named mitoxantrone resistance-associated protein (MXR), breast cancer resistance protein (BCRP) or placenta-specific ATP-binding cassette transporter (ABCP), was first cloned from drug selected human breast cancer cells MCF-7 and has been reported to transport chemotherapeutic agents including rhodamine 123, doxorubicin, mitoxantrone, topotecan and imatinib [2], [7]–[11]. More recently, ABCG2 is considered as a molecular determinant of the side-population phenotype and ABCG2 expression has been shown to protect hematopoietic stem cells from cytotoxic agents [12], [13]. In fact, high expression of ABCG2 has been found in a wide variety of cancer stem cells. Enormous effort has been devoted to the development of inhibitors for ABC transporters in the hope of circumventing MDR. Three generations of MDR inhibitors have been exploited, some of which have demonstrated ability to reverse MDR. Clinical trials evaluating the potential reversal of anticancer drug resistance by some of these MDR inhibitors are underway [14].

Tyrosine kinase inhibitors (TKIs) belong to a new class of anticancer agents, which are believed to work by competing with the ATP binding site of the catalytic domain of several oncogenic tyrosine kinases, thereby blocking downstream signal pathways pivotal for cell replication, survival, metastasis and angiogenesis. Their usefulness in cancer therapy has been demonstrated in pre-clinical models and also in the clinics [15], [16]. Recently, two tyrosine kinase inhibitors, STI-571 and AG1393, have been shown to interact with human ABCB1 and ABCC1, and inhibit their transport activities [17]. Other TKIs have also been found to overcome MDR mediated by ABC transporters, including gefitinib [18], [19], erlotinib [20], and lapatinib [21]. These reports collectively suggest that TKIs may be promising MDR inhibitors.

Vandetanib (Zactima, ZD6474, AstraZeneca Pharmaceuticals, Macclesfield, UK) is an oral small molecule inhibitor of VEGFR-2 (Flk-1/KDR, vascular endothelial growth factor receptor 2), EGFR (ErbB1/Her-1, epidermal growth factor receptor), and RET (rearranged during transfection) tyrosine kinases. It displays antitumor activity by directly inhibiting tumor cell proliferation and survival via EGFR and RET inhibition, as well as tumor angiogenesis via VEGFR inhibition [22]. Recent study reported that vandetanib may interact with ABCB1 and inhibit its function [23]. This spurs intensified effort to investigate whether vandetanib can enhance the efficacy of conventional chemotherapeutic drugs via interaction with ABC transporters in MDR cancer cells.

In this study, we evaluated the efficacy of vandetanib on ABCC1- and ABCG2- mediated MDR *in vitro*. Vandetanib inhibited the transport function of ABCC1 and ABCG2 and sensitized MDR cancer cells to substrate drugs of the two transporters. Clinical trial is warranted to study the circumvention of multidrug anticancer resistance by a combination of vandetanib with other cytotoxic agents.

## Materials and Methods

### Chemicals and reagents

3-(4,5-dimethylthiazol-yl)-2,5-diphenyllapatinibrazolium bromide (MTT), vincristine, topotecan, doxorubicin, rhodamine 123, verapamil *R*-enantiomer (*R*-VRP) and fumitremorgin C (FTC) were products of Sigma Chemical Co. Vandetanib was obtained from AstraZeneca Pharmaceuticals. Dulbecco's modified Eagle's medium (DMEM) and RPMI 1640 were products of Gibco BRL.. Monoclonal antibodies against ABCB1, ABCC1 and glyceraldehyde-3-phosphate dehydrogenase (GAPDH) were products of Santa Cruz Biotechnology, Inc.. Anti-MAP Kinase 1/2 (ERK1/2), p-ERK and p-AKT antibodies were purchased from Kangchen Co. (Shanghai, China). AKT antibody was a product of Cell Signaling Technology Inc. (Danvers, MA). ABCG2 antibody was obtained from Chemicon International, Inc. Other routine laboratory reagents were obtained from commercial sources of analytical grade.

### Cell culture

The following cell lines were cultured in DMEM or RPMI 1640 containing 10% fetal bovine serum at 37°C in the presence of 5% CO_2_: the human oral epidermoid carcinoma MDR cell line KBV200 which is ABCB1-overexpressing [23], the human epidermoid carcinoma cell line KB-3-1 and its doxorubicin-selected derivative ABCC1-overexpressing cell line C-A120 [24]; the human colon carcinoma cell line S1 and its mitoxantrone-selected derivative ABCG2-overexpressing cell line S1-M1-80 [25].

### Cell proliferation assay

MTT assay was used to assess anti-proliferation activity [26]. Briefly, cells were seeded in 96-well plates and allowed to attach overnight. To determine the cytotoxicity of vandetanib, the drug at various concentrations was added to the cells and the cells were incubated at 37°C for 68 h. MTT (5 mg/mL, 20 µL/well) was then added to the cells for 4 h (37°C). Afterwards, the formazan product from metabolism of MTT was solubilized in DMSO (200 µL/well). Optical density was measured at 540 nm, with background subtraction at 655 nm, using the Model 550 Microplate Reader (BIO-RAD, Hercules, CA, USA). The concentration required to inhibit cell growth by 50% (IC_50_) was calculated from survival curves using the Bliss method [27]. As an indirect method to assess whether vandetanib is substrate of ABCC1 and ABCG2, the cytotoxicity assays were repeated by treating the cells at various concentration of vandetanib with or without specific inhibitors of the two transporters (ABCC1: *R*-VRP 5 µmol/L) or (ABCG2: FTC 0.5 µmol/L). To test the effect of vandetanib on the chemosensitivity of MDR cancer cells, vincristine (KB-3-1 and its ABCC1-overexpressing derivative) or topotecan (S1 and its ABCG2-overexpressing derivative) at various concentrations were added to the cells with or without the addition of vandetanib. The fold-reversal factor of MDR was calculated by dividing the IC_50_ for the cells to vincristine or topotecan in the absence of vandetanib by that obtained in the presence of vandetanib.

### Doxorubicin and rhodamine 123 accumulation

The effect of vandetanib on the accumulation of doxorubicin and rhodamine 123 was measured by flow cytometry as previously described [28]. Briefly, 5×10^5^ cells were incubated in 6-well plates and allowed to attach overnight. The cells were treated with desired concentrations of vandetanib or the vehicle (DMSO) at 37°C for 3 h. Then 10 µmol/L doxorubicin or 5 µmol/L rhodamine 123 was added and the cells were further incubated for another 3 h or 0.5 h, respectively. Cells were then collected and washed twice with ice-cold PBS buffer. Finally, the cells were resuspended in PBS buffer for flow cytometric analysis (Beckman Coulter, Cytomics FC500, USA) and 1×10^4^ cells were counted. *R*-VRP was used as a control inhibitor in KB-3-1 and C-A120 cells whereas FTC was used in S1 and S1-M1-80 cells [23], [29].

### Preparation of cell lysates and western blot analysis

After vandetanib treatment, cells were harvested and rinsed twice with ice-cold PBS buffer. Cell extracts were collected in cell lysis buffer (1× PBS, 1% Nonidet P-40, 0.5% sodium deoxycholate, 0.1% SDS, 100 µg/mL phenylmethylsulfonyl fluoride, 10 µg/mL aprotinin, 10 µg/ml leupeptin). Equal amounts of cell lysate from various treatments (100 µg of protein) were resolved by sodium dodecyl sulfate polycrylamide gel electrophoresis and then electrophoretically transferred onto polyvinylidene fluoride membranes. After blocked in 5% non-fat milk in TBST buffer (10mmol/L Tris-HCL, 150mmol/L NaCl, and 0.1% Tween20, pH 8.0) for 2 h at room temperature, the membranes were incubated with appropriately diluted primary antibodies overnight at 4°C. The membranes were then washed thrice with TBST buffer and incubated with HRP-conjugated secondary antibody at 1∶5000 dilution for 2 h at room temperature. After washed thrice with TBST buffer, the protein-antibody complex were visualized by the enhanced Phototope TM-HRP Detection Kit (Cell Signaling, USA) and exposed to Kodak medical X-ray processor (Carestream Health, USA). GAPDH was used as the loading control.

### Reverse transcription-PCR

After vandetanib treatment, total cellular RNA was isolated by Trizol Reagent RNA extraction kit following manufacturer's instruction (Molecular Research Center, USA). The first strand cDNA was synthesized by Oligo dT primers with reverse transcriptase (Promega Corp.). PCR primers were 5′-cta cct cct gtg gct gaa tct g-3′ (forward) and 5′-cat cag ctt gat ccg att gtc t-3′ (reverse) for ABCC1; 5′-tgg ctg tca tgg ctt cag ta-3′ (forward) and 5′-gcc acg tga ttc ttc cac aa-3′ (reverse) for ABCG2; and 5′-ctt tgg tat cgt gga agg a-3′ (forward) and 5′-cac cct gtt gct gta gcc-3′ (reverse) for GAPDH, respectively. Using the GeneAmp PCR system 9700 (PE Applied Biosystems, USA), reactions were carried out at 94°C for 2 min for initial denaturation, and then at 94°C for 30 s, 58°C for 30 s, and 72°C for 1 min. After 35 cycles of amplification, additional extensions were carried out at 72°C for 10 min. Products were resolved and examined by 1.5% agarose gel electrophoresis. Expected PCR products were 151 bp for ABCC1, 235 bp for ABCG2, and 475 bp for GAPDH, respectively.

### Statistical analysis

All experiments were repeated at least three times. Microsoft Office Excel 2003 and the statistical software SPSS16.0 were used in data processing and analyzing the significance using the student's *t*-test. All P-values were two-sided, and the statistical significance was determined at *P*<0.05.

## Results

### ABCC1 and ABCG2 do not confer resistance to vandetanib

**Figure 1:**
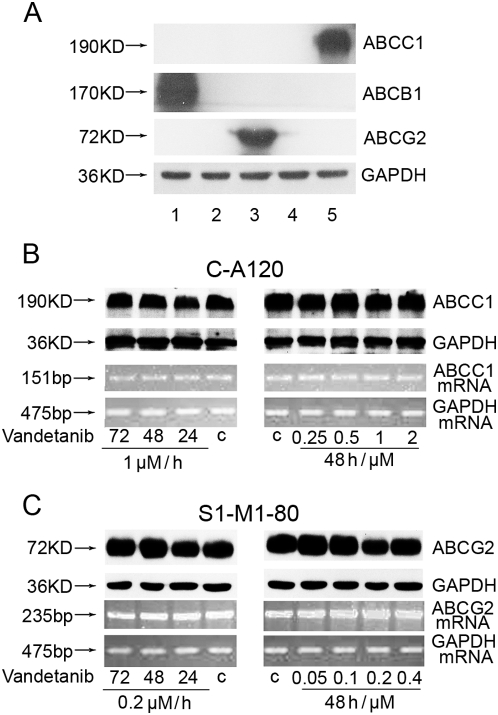
Effect of vandetanib on the expression of ABCC1 and ABCG2 in MDR cells. The protein expression of ABCB1, ABCC1 and ABCG2 in KBV200 (lane 1), S1 (lane 2), S1-M1-80 (lane 3), KB-3-1 (lane 4) and C-A120 (lane 5) cell lines without treatment was measured by Western blot analysis (A). C-A120 (B) and S1-M1-80 cells (C) were treated with vandetanib at indicated concentrations for 24, 48, or 72 h. The expression of ABCC1 and ABCG2 at both protein and mRNA level were detected as described in “Materials and Methods”. A representative result is shown from at least thee independent experiments.

**Figure 2:**
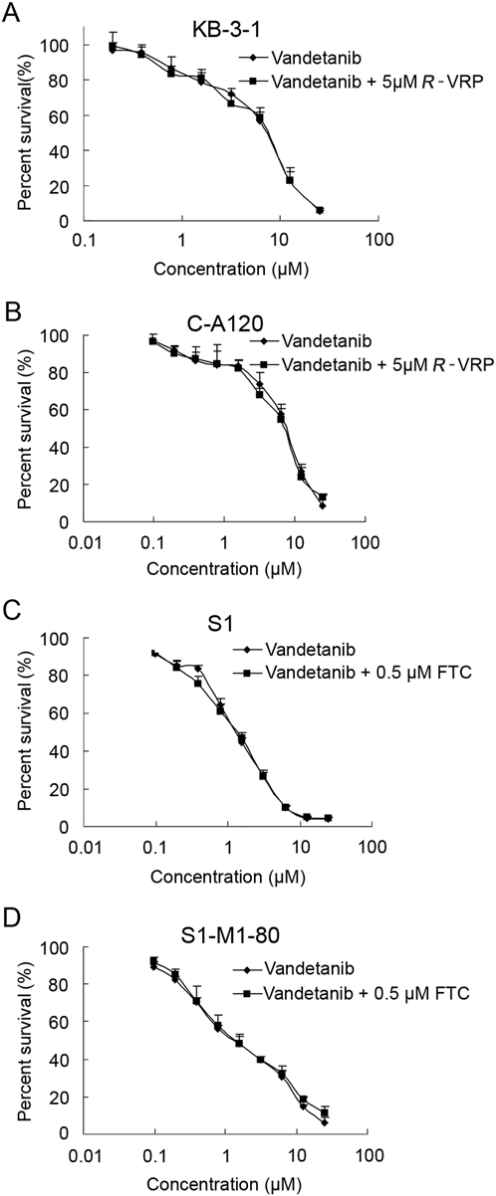
Cytotoxicity of vandetanib in the sensitive parental and drug-resistant cancer cell lines. MTT cytotoxicity assay was used to measure cell survival in KB-3-1 (A), C-A120 (B), S1 (C) and S1-M1-80 cells treated with vandetanib alone or in combination with ABCC1 inhibitor *R*-VRP or ABCG2 inhibitor FTC, as described in “Materials and Methods”. Data points represent the means±SD of at least three independent experiments performed in triplicate.

C-A120 and S1-M1-80 are drug resistance models with overexpression of ABCC1 and ABCG2, respectively (**Figure. 1A**). The basal expressions of ABCC1 and ABCG2 in the parental cell lines were nearly undetectable. MTT assay was performed to examine the cytotoxic effect of vandetanib on these MDR cells. Vandetanib showed moderate cytotoxic effect on both of the two pairs of parental and resistant cell lines (**Figure. 2** and **Table 1**). No significant difference in the cytotoxicity of vandetanib was observed between the parental and resistant cells. The IC_50_ values of vandetanib alone for KB-3-1, C-A120, S1 and S1-M1-80 cells were 4.198, 4.235, 1.291, and 1.398 µmol/L, respectively. Cytotoxicity assays were repeated with a combination of vandetanib with inhibitors of ABCC1 (*R*-VRP; 5 µmol/L) or ABCG2 (FTC; 0.5 µmol/L) in the parental and resistant cells of KB-3-1 and S1, respectively. The drug combination did not change the cytotoxic effect of vandetanib (**Figure. 2** and **Table 1**). Thus, overexpression of ABCC1 and ABCG2 do not confer resistance to vandetanib. Moreover, vandetanib is unlikely to be a substrate for ABCC1 and ABCG2.

**Table 1:** Effect of vandetanib on reversing ABCC1- and ABCG2-mediated drug resistance

Compounds	IC_50_±SD µmol/L (fold-reversal)	
	KB-3-1	C-A120
Vandetanib	4.198±0.175 (1.00)	4.235±0.419 (1.00)
+5 µmol/L *R*-VRP	4.553±0.152 (0.92)	4.522±0.776 (0.94)
Vincristine	0.003409±0.000169 (1.00)	0.654±0.040 (1.00)
+0.25 µmol/L vandetanib	0.003476±0.000336 (0.98)	0.352±0.025 (1.86)*
+0.5 µmol/L vandetanib	0.003394±0.000250 (1.00)	0.209±0.023 (3.13)**
+1 µmol/L vandetanib	0.003382±0.000394 (1.01)	0.087±0.013 (7.52)**
+5 µmol/L *R*-VRP	0.003147±0.000104 (1.08)	0.069±0.010 (9.49)**
	S1	S1-M1-80
Vandetanib	1.291±0.041 (1.00)	1.398±0.088 (1.00)
+0.5 µmol/L FTC	1.229±0.118 (1.05)	1.410±0.251 (0.99)
Topotecan	0.282±0.020 (1.00)	11.116±0.635 (1.00)
+0.05 µmol/L vandetanib	0.259±0.023 (1.09)	5.344±0.310 (2.08)*
+0.1 µmol/L vandetanib	0.269±0.044 (1.05)	2.205±0.098 (5.04)**
+0.2 µmol/L vandetanib	0.272±0.025 (1.04)	1.240±0.097 (8.96)**
+0.5 µmol/L FTC	0.277±0.029 (1.02)	0.905±0.066 (12.3)**

Cell survival was determined by MTT assays as described in “Materials and Methods”. Data are the means±standard deviation (SD) of at least three independent experiments performed in triplicate. The fold-reversal of MDR was calculated by dividing the IC50 for cells with the anticancer drugs in the absence of vandetanib by that obtained in the presence of vandetanib.

* and ** represent *P*<0.05 and *P*<0.01, respectively, for values versus that obtained in the absence of vandetanib.

### Vandetanib sensitizes ABCC1- and ABCG2-overexpressing cells to chemotherapeutic drugs

As shown in **Figure. 2**, more than 90% of cells survived when treated with vandetanib alone up to 1 µmol/L in KB-3-1 and C-A120 cells, and up to 0.2 µmol/L in S1 and S1-M1-80 cells. Therefore, vandetanib at a concentration of up to 1 µmol/L (in KB-3-1 and C-A120) or 0.2 µmol/L (in S1 and S1-M1-80) was chosen for combination treatment with known ABCC1 (vincristine) or ABCG2 (topotecan) substrate anticancer drugs. The IC_50_ values of vincristine or topotecan in the two pairs of parental and MDR cell lines when treated alone or in combination with different concentrations of vandetanib are shown in **Table 1**. *R*-VRP and FTC (inhibitor for ABCC1 and ABCG2, respectively) were used in place of vandetanib as positive control to confirm the mechanism of drug resistance in the MDR cell line models. In ABCC1-overexpressing C-A120 and ABCG2-overexpressing S1-M1-80 cells, vandetanib produced a significant dose-dependent enhancement in the cytotoxicity of vincristine and topotecan, respectively (*P*<0.05). The fold-reversal of MDR by 1 µmol/L vandetanib in C-A120 cells was about 7.5, while that by 0.2 µmol/L vandetanib in S1-M1-80 cells was about 9.5, suggesting that vandetanib is more potent in reversing ABCG2-mediated MDR. As a control, vandetanib did not increase chemosensitivity in the sensitive parental cells.

### Vandetanib modulates ABCC1- and ABCG2-mediated transport

**Figure 3:**
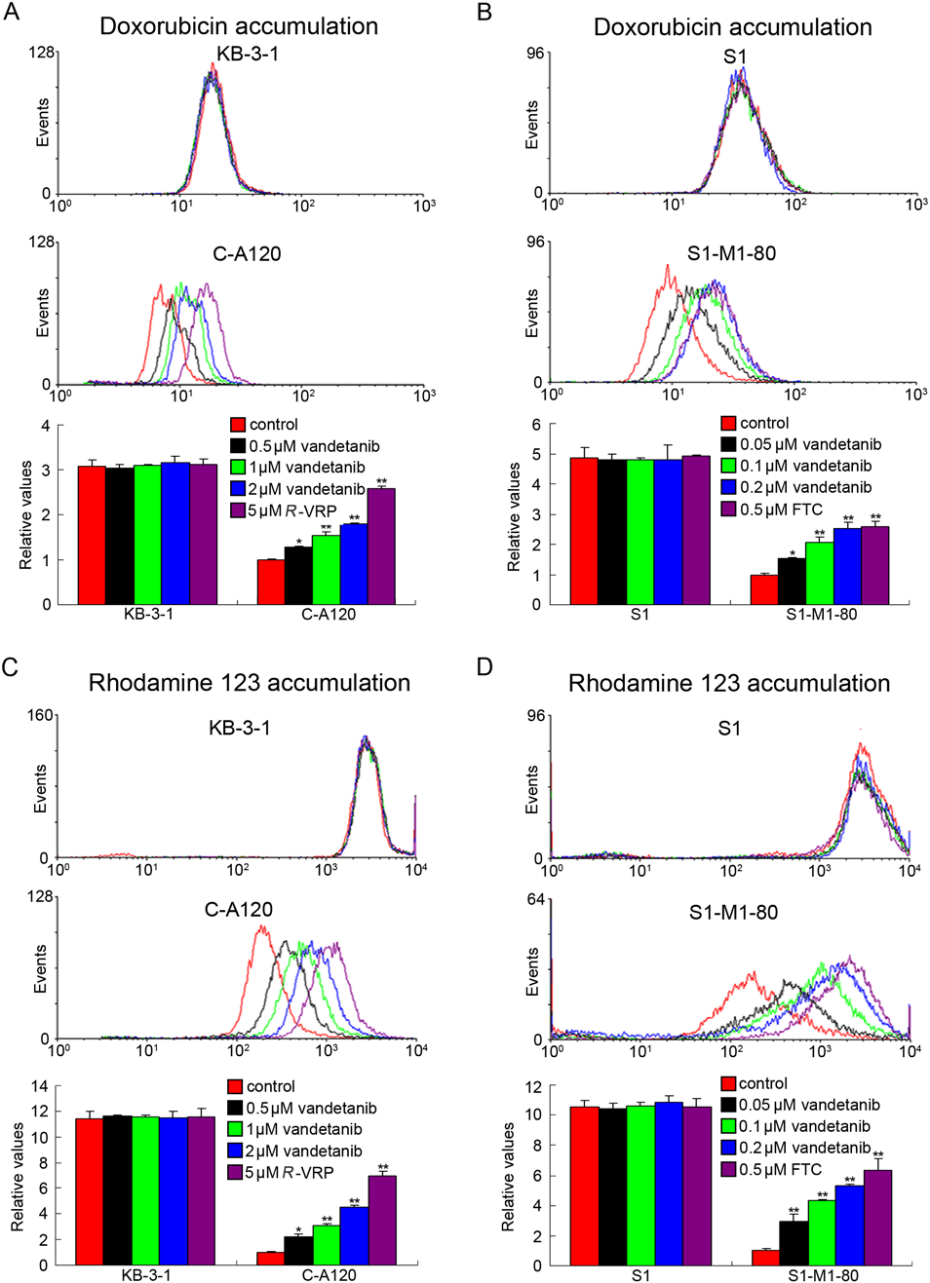
Effect of vandetanib on the accumulation of doxorubicin and rhodamine 123. The accumulation of doxorubicin (A and B) and rhodamine 123 (C and D) was measured by flow cytometric analysis as described in “Materials and Methods”. The results are presented as fold change in fluorescence intensity relative to control MDR cells. They are calculated by dividing the fluorescence intensity of each sample with that of MDR cells treated with doxorubicin or rhodamine 123 alone. Columns, means of triplicate determinations; *bars*, SD. * , *P*<0.05; **, *P*<0.01, versus the MDR control group, respectively.

The results above indicated that vandetanib could enhance the sensitivity of MDR cancer cells to certain ABCC1 and ABCG2 substrate anticancer drugs. To understand the underlying mechanisms, the intracellular accumulation of doxorubicin and rhodamine 123 in the presence or absence of vandetanib was examined by flow cytometric analysis. Upon treatment with the fluorescent substrates alone, intracellular fluorescence intensity of doxorubicin was significantly higher in the KB-3-1 (3.1-fold) and S1 cells (4.9-fold) than that in the MDR C-A120 and S1-M1-80 cells whereas that of rhodamine 123 was 11.4-fold in KB-3-1 and 10.6-fold in S1 cells compared with C-A120 and S1-M1-80 cells, respectively (**Figure. 3**). When the cells were treated in the presence of vandetanib, the intracellular accumulation of doxorubicin was 1.3-, 1.5- and 1.9-fold higher in C-A120 cells in the presence of 0.5, 1 and 2 µmol/L vandetanib, and 1.6-, 2.1- and 2.6-fold higher in S1-M1-80 cells in the presence of 0.05, 0.1 and 0.2 µmol/L vandetanib, respectively (**Figure. 3 A and B**). As shown in **Figure. 3 C and D**, vandetanib at 0. 5, 1 and 2 µmol/L increased the intracellular accumulation of rhodamine 123 by 2.2-, 3.1- and 4.6-fold in C-A120 cells, and increased the intracellular accumulation of rhodamine 123 by 3.0-, 4.3- and 5.3-fold in S1-M1-80 cells at the concentration of 0.05, 0.1 and 0.2 µmol/L, respectively. However, no significant change in the intracellular accumulation of doxorubicin and rhodamine 123 was observed in the parental KB-3-1 and S1 cells upon combination treatment with vandetanib. Taken together, these results suggest that vandetanib is able to modulate ABCC1- and ABCG2-mediated transport in MDR cells.

### Vandetanib does not alter the expression of ABCC1 and ABCG2

The reversal of ABCC1- and ABCG2-mediated MDR can be achieved either by inhibiting their function or decreasing their expression. The effect of vandetanib on the expression of ABCC1 and ABCG2 were measured by western blot analysis and reverse transcription-PCR. ABCC1 and ABCG2 expressions were not significantly altered at the protein or mRNA level (**Figure. 1 B and C**) after treatment with vandetanib at up to 1 µmol/L and 0.2 µmol/L in C-A120 and S1-M1-80 cells, respectively. The result suggests that the reversal of MDR was not obtained through inhibition of expressions of the two transporters.

### Vandetanib dose not block the phosphorylation of AKT and ERK1/2

**Figure 4:**
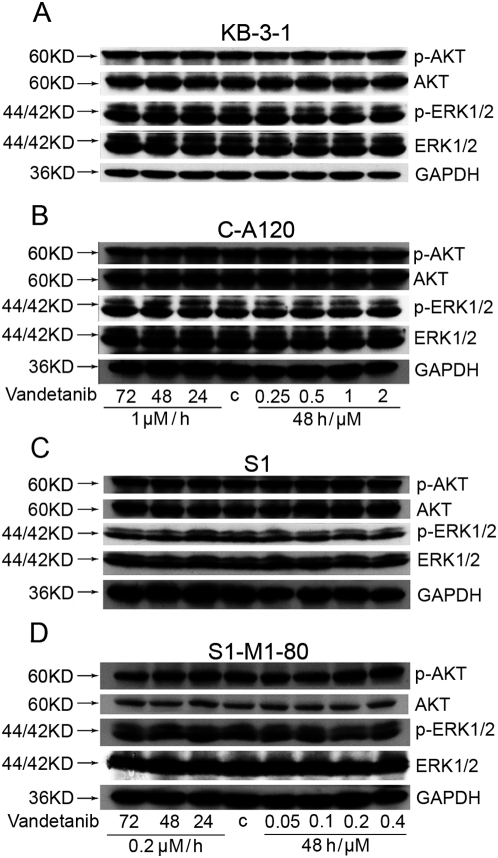
Effect of vandetanib on the blockade of AKT and ERK1/2 phosphorylation. KB-3-1 (A), C-A120 (B), S1 (C) and S1-M1-80 cells (D) were treated with vandetanib at indicated concentrations for 24, 48, or 72 h. The expressions of AKT, pAKT, ERK1/2 and pERK1/2 were examined by Western blot analysis, with GAPDH as the loading control as described in “Materials and Methods”. Independent experiments were performed at least three times and result from a representative experiment is shown.

As common downstream markers of most cytokine-receptor signal transduction pathways, the phosphorylation of AKT and/or ERK1/2 is usually used to indicate the involvement of these pathways in the activity of the tested compounds. To determine whether the MDR reversal activity of vandetanib was related to the change of AKT and ERK1/2, total and phosphorylated forms of AKT and ERK1/2 were measured after treatment with vandetanib at concentrations used in the MTT assays. As shown in **Figure. 4** **and** **S1**, after vandetanib treatment, total and phosphorylated AKT and ERK1/2 were not significantly changed in all the cell lines tested. The results suggest that the MDR reversal effect by vandetanib in MDR C-A120 and S1-M1-80 cells is independent of the inhibition of AKT and ERK1/2 phosphorylation.

## Discussion

Since Gleevec (imatinib mesylate, STI571) was approved by the FDA as first-line treatment for chronic myeloid leukemia with the oncoprotein BCR-ABL expression in 2001, molecular targeted therapy for various cancers has become an area of intense basic science and clinical research. Cytokine receptor signal transduction pathways are important mediators of cancer cell oncogenesis, proliferation, maintenance and survival. Among them, EGFR and VEGFR-2 pathways, important in cancer cells and cancer-associated endothelial cells, respectively, are the most extensively studied [30]–[32]. Different approaches have been exploited to inhibit these two pathways including monoclonal antibodies targeting EGFR and VEGFR, VEGF Trap, and TKIs [16], [33]. Currently, the potential anticancer activity of a number of TKIs, including gefitinib, erlotinib, EKB-569, EKB-569, HKI-272, CI-1033, and vandetanib, are studied in different stages of clinical trials [15].

Vandetanib (Zactima, ZD6474) is an oral small molecule inhibitor of VEGFR-2, EGFR, and RET tyrosine kinases. In vitro studies demonstrated its selective inhibition on VEGFR-2, EGFR, VEGFR-3 and RET tyrosine kinase activities, with IC_50_ of 40, 110, 500 and 100 nmol/L, respectively [34], [35]. Vandetanib has been shown to inhibit the proliferation of vascular endothelial growth factor-A -stimulated human umbilical vein endothelial cell *in vitro* (IC_50_ 60 nmol/L). Further studies demonstrated that it inhibits proliferation and induce apoptosis of cancer cells in vitro and that it also inhibits angiogenesis and progression of many xenograft and orthotropic nude mice models, including non-small cell lung cancer [36], small-cell lung cancer [37], gastric cancer [38], nervous system tumors [39], pancreatic cancer [40], colon cancer [41], hepatocellular carcinoma [42], and prostate cancer [43], via inhibiting phosphorylation of AKT and/or ERK1/2. Unlike other chemotherapeutic agents that readily develop cross-resistance towards other structurally related or unrelated compounds, vandetanib has not been reported to develop cross-resistance to prior chemotherapeutic agents, either *in vivo* or *in vitro* [44]. Phase I studies showed that vandetanib is generally well tolerated at dose up to 300 mg/day, with a once-daily oral administration schedule [45], [46]. Phase II evaluation in patients with advanced refractory NSCLC demonstrated improvements in progression-free survival both as monotherapy (versus gefitinib) and in combination with docetaxel (versus docetaxel alone) [47]. These positive outcomes have led to the initiation of Phase III trials of vandetanib in advanced NSCLC. Clinical development is also ongoing in other tumor types and encouraging evidence of antitumor activity has been reported [47].

ABC transporters-mediated MDR is a major obstacle to cancer chemotherapy. Quinazolines are some of the most promising inhibitors of receptor tyrosine kinases by competition with ATP for ATP-binding site [48]. Many TKIs with core structure of quinazolines have been demonstrated to inhibit the function of ABC transporters and reverse MDR, including gefitinib and erlotinib [19], [20]. Vandetanib shares the same quinazoline moiety with other TKIs, evoking the possibility that it can also interact with ABC transporters. A recent report has demonstrated the interaction between vandetanib and ABCB1 [23].

In the present study, our data showed that overexpression of ABCC1 and ABCG2 confer drug resistance to the drug-selected resistant sublines C-A120 and S1-M1-80, respectively. C-A120 cells were about 190-fold resistant to vincristine compared with its parental KB-3-1 cell line whereas S1-M1-80 was about 40-fold resistant to topotecan compared with its parental S1 cell line (**Table 1**). Both of the two resistant cell lines had similar sensitivity to vandetanib compared with sensitive parental cells. More importantly, co-incubation with ABCC1 inhibitor *R*-verapamil or ABCG2 inhibitor fumitremorgin C did not change the sensitivity of the tested cells to vandetanib, strongly implying that overexpression of functional ABCC1 and ABCG2 dose not confer resistance to vandetanib, and that vandetanib is not a substrate of ABCC1 and ABCG2. The latter property probably explains why vandetanib is devoid of cross-resistance to prior chemotherapeutic agents in clinical trials [44]. Our data also demonstrated the ability of vandetanib to enhance cytotoxicity of known ABCC1 substrate vincristine and ABCG2 substrate topotecan in MDR cells (**Table 1**, *P*<0.05). Vandetanib at 1 µmol/L significantly increased the sensitivity of C-A120 cells to vincristine by 7.5-fold and 0.2 µmol/L of vandetanib enhanced the sensitivity of S1-M1-80 cells to topotecan by 9-fold. While vandetanib at 0.2 to 1 µmol/L only affect cell proliferation very minimally, the two concentrations are readily attainable clinically [46] (**Figure. 2**). As a control, vandetanib at the same concentrations did not have any significant effect on the sensitive parental cells, suggesting that the sensitization of the resistant cells by vandetanib is due to its specific effect on ABCC1 and ABCG2.

To investigate the mechanism of ABCC1- and ABCG2-mediated MDR reversal by vandetanib, ABCC1 and ABCG2 expression and transport activity were examined. Consistent with the overexpression and therefore higher transport function of ABCC1 and ABCG2, C-A120 and S1-M1-80 cells had lower intracellular accumulation of doxorubicin and rhodamine 123, respectively (**Figure. 3**). Vandetanib treatment significantly increased the accumulation of doxorubicin and rhodamine 123 in a dose-dependent manner (*P*<0.05). However, no significant change was found in the parental KB-3-1 and S1 cells. Taken together, these data strongly advocate that vandetanib can inhibit the transport function of ABCC1 and ABCG2, thereby increasing the intracellular concentration of their substrate chemotherapeutic drugs. However, vandetanib treatment did not change the expression of ABCC1 and ABCG2 at both protein and mRNA level in the cells (**Figure. 1** **B and C**). It has been reported that activation of PI3K/AKT and/or ERK pathways is related to resistance to conventional chemotherapeutic agents [49]–[51]. On the other hand, vandetanib treatment is associated with AKT and/or ERK1/2 inhibition *in vitro* or *in vivo*. However, our data revealed no significant alteration of the phosphorylation of AKT and ERK1/2 in the tested cell lines (**Figure. 4** **and** **S1**), suggesting that inhibition of AKT and ERK1/2 is not involved in the reversal of ABCC1 or ABCG2-mediated MDR by vandetanib.

The binding of ATP to the nucleotide-binding site of ABC transporter is essential for substrate transport, and the hydrolysis of ATP by ATPase activity of the transporter is critical for restoring the transporter to its active conformational state [52]. Inhibition of ABC transporter activity by modulators can be achieved by blockage of (1) specific recognition of the substrate, (2) binding of ATP, (3) ATP hydrolysis, or (4) coupling of ATP hydrolysis to translocation of the substrate [14]. The drug efflux function of ABCB1 and ABCG2 is coupled to ATP hydrolysis which is stimulated in the presence of ABCB1 and ABCG2 substrates. Based on their effect on ATPase activity of ABC transporters, compounds (anticancer drugs and MDR reversing agents) can be categorized into three distinct classes. The first class of compounds stimulates ATPase activity at low concentrations but inhibits the activity at high concentrations, the second class of compounds enhances ATPase activity in a dose-dependent manner without any inhibition, whereas the third class of compounds inhibits both basal and stimulated ATPase activity [53]. Erlotinib and lapatinib, two other tyrosine kinase inhibitors, have been demonstrated to reverse ABCB1- and ABCG2-mediated MDR and can stimulate the ATPase activities of the transporters at low concentrations [20], [21]. It has been reported that vandetanib can directly interact with ABCB1 and increase the ATPase activity of ABCB1 in a dose-dependent manner [23]. This suggests that vandetanib belongs to the second class of compounds to interact with ABC transporters.

The concept of cancer stem cells, suggesting that cancers originate and are maintained by a rare fraction of cells with stem cell properties, has played a pivotal role in changing our view of carcinogenesis and chemotherapy [14]. Recent studies suggested that cancer stem cells may be responsible for tumorigenesis and contribute to resistance to cancer chemotherapy [14], [54]. ABCG2 is found to be expressed in a wide variety of cancer stem cells and may be responsible for their drug resistance phenotype [55]. Our data showed that vandetanib was able to inhibit ABCG2 transport activity and to reverse MDR even at low concentration. It could be used in conjunction with other conventional anticancer drugs to eradicate the cancer stem cells.

In conclusion, vandetanib reverses ABCC1- and ABCG2-mediated MDR by directly inhibiting ABCC1 and ABCG2 function at clinically relevant concentrations, resulting in an increase of intracellular concentrations of substrate chemotherapeutic drugs to the two transporters. The MDR reversal seems to be independent of the blockade of tyrosine kinases. Our results suggest that vandetanib can be used in conjunction with conventional ABCC1 and ABCG2 substrate anticancer drugs to combat the problem of multidrug resistance in the clinic.

## Supporting Information

Figure S1 (0.16 MB PDF)
