# Supplementary material for: Vandetanib (Zactima, ZD6474) Antagonizes ABCC1- and ABCG2-Mediated Multidrug Resistance by Inhibition of Their Transport Function
Source: PLoS One. 2009 Apr 23;4(4):e5172. doi: 10.1371/journal.pone.0005172 (PMC2669214; doi:10.1371/journal.pone.0005172)
Supplement: Figure S1 — (0.16 MB PDF) [file pone.0005172.s001.pdf]

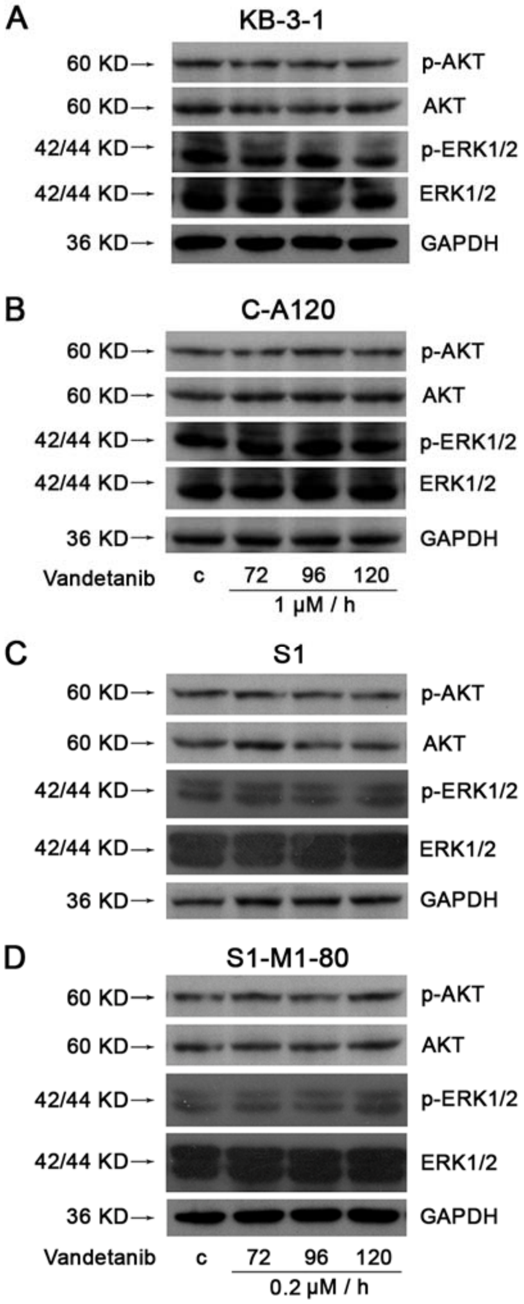

Figure S1. Effect of vandetanib on the blockade of AKT and ERK1/2 phosphorylation for long term.

KB-3-1 (A), C-A120 (B), S1 (C) and S1-M1-80 cells (D) were treated with vandetanib at indicated concentrations for 72, 96, or 120 h. The expressions of AKT, pAKT, ERK1/2 and pERK1/2 were examined by Western blot analysis, with GAPDH as the loading control as described in “Materials and Methods”. Independent experiments were performed at least three times and result from a representative experiment is shown.
